# Video Production and Distribution Platform in Swiss Sports Teams: An Analysis of Acceptance and Willingness to Pay

**DOI:** 10.3389/fspor.2021.722043

**Published:** 2021-09-24

**Authors:** Marcel Huettermann, Fabian Haefliger, Valerio Stallone

**Affiliations:** ^1^Institute for Marketing Management, ZHAW School of Management and Law, Winterthur, Switzerland; ^2^Swisscom Broadcast AG, Swisscom Event and Media Solutions, Zurich, Switzerland

**Keywords:** video production, distribution platform, sport, acceptance, willingness-to-pay

## Abstract

Swisscom Asport (“Asport”) has set itself the target of covering the entire video process from production to distribution. Its services should be affordable not only for professional sports clubs but also for their amateur counterparts. Despite limited resources, clubs want to increase their attractiveness through new technologies and meet members' needs. This paper examines the factors that lead to the acceptance of Asport services by sports clubs. In addition, the willingness to pay for these services is evaluated. Pricing is critical to success in innovation and also because of the limited financial resources of sports clubs. Based on the literature research, a conceptual model was developed based on the unified theory of acceptance and use of technology model (UTAUT), tested using an online survey of Swiss amateur football clubs, and evaluated using regression analysis. The study findings show that social environment exerts the strongest influence on behavioral intention, defined as “acceptance” in this paper. Furthermore, the two independent variables, performance expectancy and effort expectancy, have a significant effect on user acceptance. In contrast to the original model (UTAUT), this paper demonstrates the direct influence of attitude to use. Of the independent variables, facilitating conditions have an additional effect on attitude to use. The results demonstrate that it is not acceptance but attitude to use that influences willingness to pay. An in-depth evaluation of willingness to pay shows that the optimal price point is 83.3% lower than the price offered by the company; however, there are budget-dependent variations in the assessment.

## Introduction

Digitalization is fundamentally changing the world and our lives in every respect, whether through the way we interact or the range of new services (Dellea et al., 2014). Changes in the digital world take place many times faster, and much less time passes from introducing new technology to achieving normality (Dellea et al., 2014). Consumer behavior has also adapted accordingly, and customers want access to goods and services anytime and anywhere. This effect is reinforced by the fact that most consumers today are digital natives, i.e., people who have grown up with digital technologies (Dellea et al., 2014). These sweeping changes are also transforming the entire sports industry including the way sports are played, consumed, and analyzed (Ráthonyi et al., 2018). Often the focus is on technologies that offer new opportunities for various stakeholders such as players, sports clubs, media, or sponsors (Elishkov et al., 2017). To address all these different stakeholders and their needs, a large number of often-young companies compete in the global sports market (Elishkov et al., 2017).

One company that is active in Switzerland and wants to digitize the Swiss sports video world is Swisscom Asport (“Asport”). Their product aims to cover the entire video process, from the production of sporting events to distribution, in a simple and automated way, with the help of the latest technology (Asport, 2020; Swisscom Asport, 2020). Asport wants to offer equal opportunities through affordable technologies and focus not only on professional sports clubs but also on their amateur counterparts (Asport, 2020). Amateur clubs often face various challenges such as recruiting members, finding volunteers, raising funds, and marketing through sponsors (Winand et al., 2016). At the same time, they are concerned with the social changes caused by digitalization and the resulting needs of members who do not want to miss out on the use and possibilities of technology in their sports club (Volkmann et al., 2019). Asport services are designed to assist with these challenges (Asport, 2020). For example, the use of technology can increase the attractiveness of the club, especially for the younger generation (Volkmann et al., 2019). Furthermore, the recording and online publication of matches provides the club with new opportunities to market itself and generate additional revenue (Asport, 2020). For Asport to successfully establish its services on the market in the long term and further optimize them, it is crucial to understand how these products function. This also includes knowledge about the factors that lead to acceptance or rejection by users. These questions are addressed in research on technology acceptance, which is widely covered in the literature (Reichwald, 1982). Another success-critical aspect that mainly determines the profitability of a company is pricing. The basis for this is an understanding of consumer willingness to pay (Völckner, 2006). This knowledge seems even more important for Asport owing to the limited financial resources of amateur sports clubs. After all, price is the determining factor in whether clubs can afford new technology to serve their members (Volkmann et al., 2019).

The aim of this paper is to determine the factors that lead to the acceptance of Asport services and analyze the willingness to pay for Asport. These two topics will be reviewed based on the literature and tested using a quantitative method, based on a survey carried out among Swiss amateur football clubs.

The following research questions can be derived from the objectives:

1. *What factors influence the acceptance of Asport services by amateur sports clubs?*
2. *Is an amateur sports club's willingness to pay influenced by its acceptance of Asport services?*
3. *How high is the willingness to pay for Asport services among sports clubs in the amateur sector?*

While there are existing studies that apply technology adoption in the context of sports technologies (Hur et al., 2011; Kwak and McDaniel, 2011; Ibrahim, 2014; Chien-Ta and Chao-Hsiang, 2015; Byun et al., 2018; Kim and Chiu, 2019), there are none using UTAUT. Moreover, all these studies focus on the individual consumer rather than the sports club. For instance, the study by Kwak and McDaniel (2011) employed the Technology Acceptance Model (TAM; Davis et al., 1989) as a theoretical framework to investigate fantasy sports league consumption. Their findings show that domain-specific knowledge, ease of use, social support and gender positively influence favorable beliefs and behavioral intentions toward a particular technology system, which is in line with previous TAM research (Alshare et al., 2005; Ha et al., 2007; Zhang and Mao, 2008). Factors influencing consumers' intention and actual behavior in using sports brand apps were examined using TAM by Byun et al. (2018). The results indicate that the level of enjoyment had a significant positive influence on perceived ease of use, while perceived ease of use also positively influenced perceived usefulness. In contrast, we focused on club managers instead of individual consumers and used the UTAUT model instead of TAM. While TAM is considered a robust model, it has been criticized for being too parsimonious to explain complex psychological processes such as behavior and technology acceptance (Venkatesh et al., 2003).

Acceptance research is located in social science-related studies, and it investigates the background of user acceptance or rejection of innovations (Reichwald, 1982). The aim is to explore the influence of an innovation's design on the end-user intention to adopt it and explore the interrelation between innovation introduction and its resulting impact (Reichwald, 1982). In the context of business economics, acceptance research is relevant in the development of the organization, the introduction of information systems, and in marketing theory—as well as having a differentiating significance in corresponding areas (Simon, 2001). Organizational theory examines how acceptance by members of an organization changes when decisions are enforced and structures are changed (Mühlen, 1998). In the area of marketing, it examines the acceptance or rejection of new services and products (Kollmann, 1998). Business informatics combines these two approaches because information systems are new products and often result in organizational adjustments when they are introduced (Lucas, 1975).

In applied research, Van Westendorp's price sensitivity measure is often used to test willingness to pay (Roll et al., 2010). For example, the European market research institute, GfK, uses this method to determine critical price points for new products (Breidert et al., 2006). As early as 1976, Van Westendorp noted that price was a relevant factor in research circles, but this was not reflected in the number of techniques used to determine the optimal price. Therefore, he introduced his price sensitivity meter (PSM) method, which belongs to the direct customer survey (Van Westendorp, 1976). The underlying idea is that each product has a specific price-setting range. If it falls below that range, the product is perceived as inferior quality, but when exceeded, the consumer will not purchase it because it represents poor value for money (Diller, 2008). This method aims to determine the optimal price and an acceptable price range based on four questions (Van Westendorp, 1976).

After introducing a product to potential customers, the following four questions are asked (Van Westendorp, 1976):

- “At which price on this scale are you beginning to experience …… {test-product) as cheap?”
- “At which price on this scale are you beginning to experience …… {test-product) as expensive?”
- “At which price on this scale you are beginning to experience …… {test-product) as too expensive—so that you would never consider buying it yourself?”
- “At which price on this scale you are beginning to experience …… {test-product) as too cheap—so that you say at this price the quality cannot be good?”

The results can be shown in an example diagram, the answers to the individual questions being aggregated and presented accordingly (Wildner, 2003). To determine the acceptable price range, the inverses of the “cheap” and “expensive” curves are formed and renamed “not cheap” and “not expensive” (Reinecke et al., 2009). The intersection of the two “not cheap” and “too cheap” curves is the price lower limit. This makes sense since an additional reduction in price would cause the proportion of those who find the product too cheap to exceed the proportion of those who do not consider it cheap (Reinecke et al., 2009). The price ceiling is determined by the intersection of the “too expensive” and “not expensive” curves and is also described as the point of marginal expensiveness. An increase in the price does not make sense because the proportion of people describing the product as too expensive would exceed those who do not view the product as expensive (Reinecke et al., 2009).

The optimal price point is defined by the intersection of the two curves “too cheap” and “too expensive.” At this point, the same number of potential customers think the product is either too cheap or too expensive (Khandker and Joshi, 2019). However, this does not take cost structures into account but instead gives the optimal price from a demand perspective, namely, when customer resistance to purchase is lowest (Lewis and Shoemaker, 1997). The indifference price is formed from the “expensive” and “cheap” curves and means that at this price, an equal number of respondents think that the product is either expensive or cheap (Khandker and Joshi, 2019). According to Van Westendorp (1976), this price usually represents the median price effectively paid by customers (or the price of the market leader). Moreover, the gap between the indifference price and the optimal price shows the price sensitivity of potential customers; the smaller the difference, the more sensitive the price (Reinecke et al., 2009).

According to Reinecke et al. (2009), the Van Westendorp method is especially suitable for estimating the price of innovations for which competitors and price expectations do not yet exist. In particular, the knowledge gained of the acceptable price range provides a valuable contribution to price-setting. Studies have been identified that incorporate a willingness to pay into a technology acceptance model and capture it using the Van Westendorp method. For example, willingness to pay was determined in Saha et al. (2020), using questions to establish whether the subject was willing to pay more in some instances. One statement from the survey read, “*I would continue to buy from this website if its prices increase somewhat”* (Saha et al., 2020). Arogundade et al. (2016) also asked whether or not the respondent was willing to pay more for secure software.

The basis of this work is the UTAUT model by Venkatesh et al. (2003), which is designed to test acceptability. The choice of the model is justified by the fact that it is the amalgamation and further development of various technology acceptance models. At the same time, applicability and generalizability are high and have already been used as a basis in various studies and adapted according to the context (Venkatesh et al., 2012). Determinants with a direct influence on behavioral intention are performance expectancy, effort expectancy, and social influence. Together with facilitating conditions, behavioral intention itself is a determinant with a direct influence on actual system use (Venkatesh et al., 2003). Determinants are moderated by the variables of gender, age, experience, and voluntariness of use (Venkatesh et al., 2003). Based on Venkatesh et al.'s (2003) longitudinal study and interviewing participants at different points in time, from introduction to use over time, the experience variable could be captured.

We chose gender and age as moderators for several reasons. Age was found to be an important moderator of behavioral intention to use information and communication technologies (ICT) in other studies (Venkatesh et al., 2012; Magsamen-Conrad et al., 2015). Despite being the most important predictor across all age groups, the effects of performance expectancy appear to be stronger for younger adults than for older ones (Wang and Wang, 2010; Venkatesh et al., 2012; Cimperman et al., 2016). An opposite pattern was found for the other determinants. For adults above the age of 50, it was mainly effort expectancy, social influence, and enabling conditions that influenced intention to use ICT (Venkatesh and Davis, 2000; Venkatesh et al., 2003; Cimperman et al., 2016).

Besides age, gender is a important demographic variables related to information and communication technology use and has been widely examined (Parameswaran et al., 2015). Prior studies have suggested that gender plays an important role in explaining behavioral intention in information system research (Sun and Zhang, 2006; Tarhini et al., 2014). The study by Venkatesh et al. (2003) shows that performance expectancy is the strongest factor on behavioral intention, and the effect is stronger for men and younger individuals. Further, the influence of effort expectancy is higher for women and older individuals, although it decreases with experience (Venkatesh et al., 2003). The proportion of moderators is so high for social influence that without them the relationship is not significant. In addition, social pressure decreases with experience, and if the system is used voluntarily, it is not relevant (Venkatesh et al., 2003). Facilitating conditions did not have a significant effect on behavioral intention, as this was explained by effort expectancy. However, a significant relationship exists to effective use only through the moderators of age and experience, with the effect increasing for older individuals and higher experience (Venkatesh et al., 2003).

Although it results from the examination of the eight acceptance models that seven constructs have a significant effect on behavioral intention, only the four presented are represented in the UTAUT (Venkatesh et al., 2003). Although the authors test the two variables, they formulate the hypothesis that computer self-efficacy and computer anxiety do not have a significant influence. The reason for this is a study conducted before that, which shows that the effect is entirely caused by effort expectancy (Venkatesh and Davis, 2000). One construct that has produced mixed results in past studies of technology acceptance is attitude toward use and its significance on behavioral intention. The authors of the UTAUT postulate that attitude only has a significant impact when performance and effort expectancy are not part of the determinants and accordingly exclude the construct from the model (Venkatesh et al., 2003). Empirical validation of the model confirms the assumptions and no significance is found for any of the three themed factors (Venkatesh et al., 2003).

In a meta-analysis by Khechine et al. (2016) comprising 74 publications that applied the UTAUT model, it was confirmed that performance expectancy has the strongest influence on behavioral intention. Further, as in Venkatesh et al. (2003) a significant influence of effort expectancy and social influence on behavioral intention was demonstrated. Unlike in the original model, facilitating conditions have a significant effect on behavioral intention in the study by Khechine et al. (2016) (Venkatesh et al., 2003).

In their study, Dwivedi et al. (2019) revisited the UTAUT based on a combination of a meta-analysis comprising 162 studies and a structural equation model. They were able to demonstrate that there is a direct influence from the independent variables of performance expectancy, effort expectancy, social influence, and facilitating conditions on usage attitudes, with performance expectancy having the strongest effect. Further, a significant and direct relationship was demonstrated from usage attitude to behavioral intention, again in contrast to the original model (Venkatesh et al., 2003; Dwivedi et al., 2019).

The object of this study—Asport—is neither a classic use case in the corporate context, as applied in the UTAUT model, nor in the consumer context, as in the UTAUT 2 model. From the authors' point of view, using the UTAUT as a basis is more logical because sports clubs as organizations bear the cost of the system. Accordingly, the feature of UTAUT 2 in that consumers must pay for their own use and can decide whether to purchase and use the system is no longer applicable (Venkatesh et al., 2012). However, working for an amateur sports club is not comparable to a traditional employment relationship, so use of the system must be considered voluntary. This is comparable with UTAUT 2, so the willingness to use moderator is removed from our model.

Venkatesh et al. (2003) postulated and proved that self-efficacy and anxiety have no significant effect on behavioral intention and were removed as determinants. This was criticized, at least concerning self-efficacy, by Moghavvemi et al. (2013). According to Yuen et al. (2010), users perceive new technologies as complex, and confidence in their own abilities has a relevant influence on acceptance. To address this aspect and to verify the findings of Venkatesh et al. (2003), the two independent variables were included in our model. The same applies to the attitude to use variable. In this paper, and as shown in the conceptual model below, attitude to use is placed between the independent variables and behavioral intention. The aim is to examine both the direct influence of attitude to use on behavioral intention and the effect of the independent variables on attitude to use. This is in line with the meta-analysis by Dwivedi et al. (2019), who demonstrated the direct influences described.

In this paper, unlike in the UTAUT model, actual use is not verified owing to the implementation options. Accordingly, the endpoint of the acceptance process here must be behavioral intention and is equivalent to acceptance. This application fits with the definition of acceptance in this thesis, which states that acceptance occurs when behavioral intention has been formed.

In the UTAUT model, only an influence of the facilitating conditions on actual system use was demonstrated (Venkatesh et al., 2003). Different results were obtained by Khechine et al. (2016), who demonstrated an effect on behavioral intention. In addition, the findings of Dwivedi et al. (2019) show an effect on attitude to use. Based on these findings, the conceptual model examines the relationship between facilitating conditions, attitude to use, and behavioral intention. As mentioned above, the voluntary nature of use was excluded as a moderator. The same is true for experience, as only a single measurement was performed in this study owing to time constraints. Based on the previous discussion, hypotheses are now derived.

**Figure 1:**
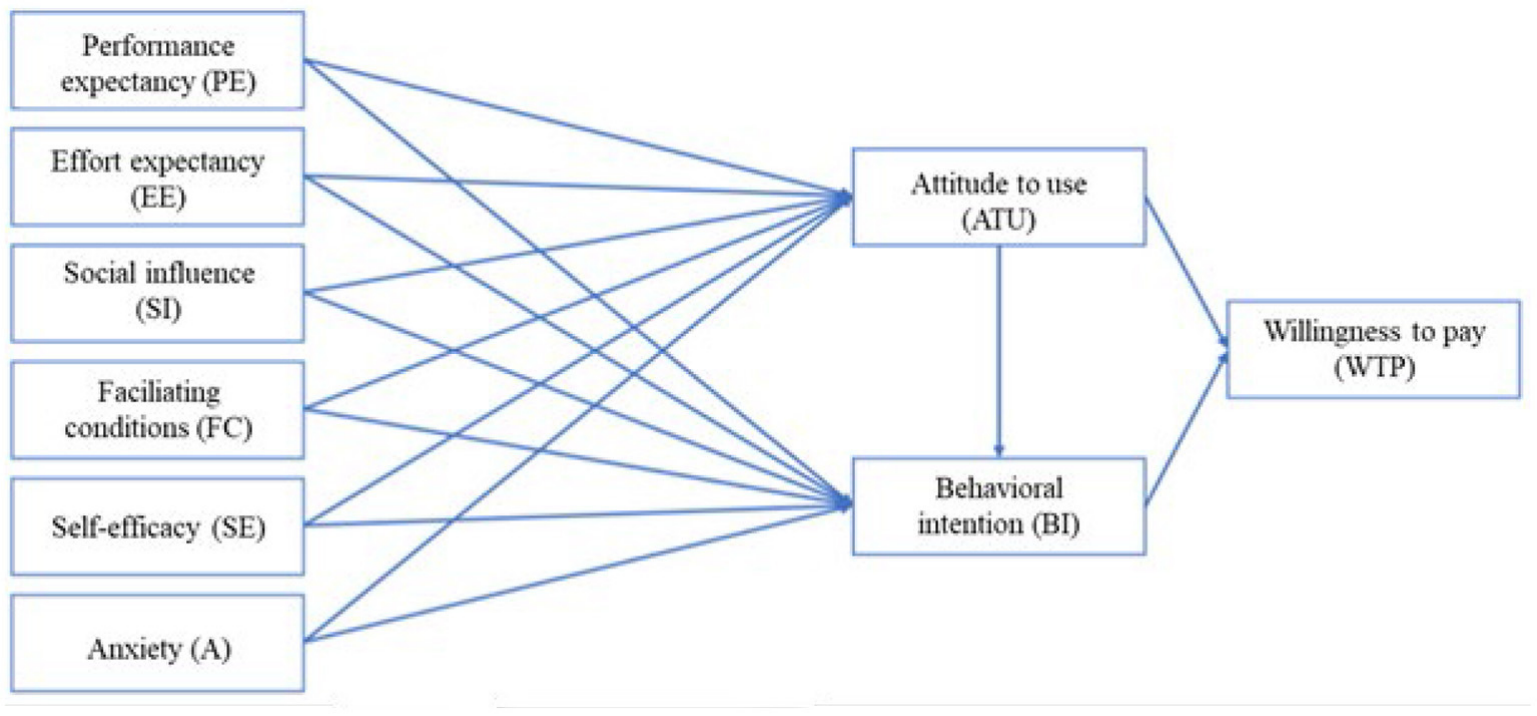
Conceptual model.

In addition to acceptance, willingness to pay is of interest and included in the conceptual model. The literature shows different approaches to this variable in technology acceptance models, and no universal implementation practice exists. Therefore, based on the context of the application, it was decided that willingness to pay should be the study endpoint. It needs to be determined whether attitude to use as well as behavioral intention or acceptance influences willingness to pay. Based on this derivation and the described adjustments, the conceptual model for this paper is shown below (Figure 1).

## Materials and Methodology

Based on Venkatesh et al. (2003), a quantitative survey was conducted. The advantage of this method is that the standardized questionnaire allows a large sample to be collected and the answers to be compared (Böhler, 2004). To obtain the largest possible number of subjects while keeping time and costs low, an online survey was conducted (Hussy et al., 2013). In many previous studies, the technology acceptance models were queried either several times during or after the completion of the acceptance process (Venkatesh et al., 2003). Due to the starting point of this study—namely that Asport only has a few customers who already use the services—the focus was placed on potential customers or users rather than actual users. This means that behavioral intention is the endpoint leading to adoption. In addition, due to the time constraints of this thesis, the survey was conducted once only.

To answer the questionnaire without having to use the system, a video was created that participants could watch as part of the survey and before answering the questionnaire. Davis (1985) used video to substitute for actual use of a system in his study and judged the method promising. The only criticism he noted was that the examination of usability—the counterpart of effort expectancy in the UTAUT model—was not optimal (Davis, 1985). The video was created in cooperation with our practice partner and included a presentation of Asport and its services. Screen recordings of the system were made, and a sample video of an automated recording was shown for the most realistic implementation possible.

### Operationalization

Existing scales were used to make the variables of the conceptual model operational, and the constructs and items for testing acceptability were questioned in line with Venkatesh et al. (2003). The willingness to pay variable was tested according to Van Westendorp (1976). The acceptance constructs were queried using multi-item scales, as these have a higher information content than single-item scales. Furthermore, the reliability of multi-item scales tends to be higher due to the lower dependence of a single item (Kuß et al., 2014). Items were reviewed using a five-point scale ranging from “strongly disagree” to “strongly agree.” This is because compared to the seven-point scale in line with Weijters et al. (2010), a five-point scale is recommended when the survey is conducted in a general population and regressions are subsequently calculated.

The following table shows the individual constructs with the respective items for the technology acceptance questionnaire based on Venkatesh et al. (2003). The items were translated into German and adapted to the context. Unlike what is shown here, items were randomized based on their similarity in the survey; according to Goodhue and Loiacono (2002), this can improve reliability (Table 1).

**Table 1:** Representation of the queried items per construct.

**Construct**	**Items**
Performance expectancy (PE)	I find the system useful for my activities in the club
	The system allows me to complete tasks faster
	Using the system increases my productivity
Effort expectancy (EE)	I find it easy to learn how to use the system
	I find it easy to use the system
	My interaction with the system is clear and understandable
	For me, it's easy to build the skills to use the system
Social influence (SI)	People in other roles in the club think I should use the system
	Players and players of the club think I should use the system
	People I care about think I should use the system
	People around me think I should use the system
Facilitating conditions (FC)	I have the necessary prerequisites to use the system
	I have the necessary knowledge to use the system
	The system is compatible with other systems used in the club
	I can get help from others if I have problems using the system
Self-efficacy (SI)	I could do a job or an activity with the system if.
	…there would be no one there to support and guide you
	…you could call someone for help if you got stuck
	… you'd have plenty of time to get a job done
	…you would only have the support function of the system to assist you
Anxiety (A)	I have reservations about using the system
	It scares me to think that I could lose a lot of data with the system if I press the wrong key
	I hesitate to use the system because of my fear of making mistakes that I cannot correct
	The system is a little intimidating to me
Attitude to use (ATU)	Using the system is a good idea
	The system makes my job at the club more interesting
	I enjoy working with the system
	I like working with the system
Behavioral intention (BI)	I intend to use the system for my function in the association
	I try to use the system for my function in the club whenever possible
	I plan to use the system on a regular basis

No consistent method was found in the literature that tests willingness to pay when the variable is part of a technology acceptance model. For example, willingness to pay was determined in Saha et al. (2020) through questions aimed at ascertaining whether the subject was willing to pay more in some instances. One question in the form of a statement read: “*I would continue to buy from this website if its prices increase somewhat”* (Saha et al., 2020, p. 11). Arogundade et al. (2016) also asked whether the respondent was willing to pay more for secure software development. In this work, however, willingness to pay is to be determined on the one hand for answering the hypotheses as well as on the other hand as itself. According to Reinecke et al. (2009), the Van Westendorp method is particularly suitable for estimating the price of innovations for which competitors and price expectations do not yet exist. This is the case with Asport, which is why a direct method was used. Willingness to pay is determined using Van Westendorp's (1976) four questions presented earlier but adapted to the context of this study. The standard evaluation of this method was conducted by aggregating the answers and comparing the results graphically for each question (Reinecke et al., 2009). In addition, to test the hypotheses, Question 2 was defined as an indicator of willingness to pay. As previously explained, this indicates the price that a respondent deemed expensive but would probably still be willing to pay. The reason for this is that hypothesis testing requires data at the level of the respondent. From the authors' point of view, this indicator is the most appropriate, as it suggests the maximum price that would be paid without losing the behavioral intention to buy.

### Selection of Subjects

All the e-mail addresses of Swiss football clubs were available to the authors of this paper. As the focus here was on amateur clubs, those from the Raiffeisen Super League (1st division) and Brack.ch Challenge League (2nd division) were excluded. As the survey was produced in German, only football clubs in German-speaking regions were included. Since the survey was intended to test acceptance by potential users, those in the football club who would use such a system to carry out their duties were also written to individually—for example, board directors or those otherwise active in the sports sector. Accordingly, the survey population comprises all potential system users at amateur football clubs in German-speaking regions of Switzerland that play in the Promotion League or at a lower level.

The survey was sent by e-mail to the generic addresses of 797 football clubs as well as the personal accounts of those active at a senior management level or having a sports-related role. To further increase the number of participants, a reminder e-mail was sent out. In order to be able to answer the questionnaire without using the system, a video was created which the participants could watch directly in the survey and before answering the questionnaire. Davis (1985) already used video as a substitute for the effective use of a system in his study and judges the method as promising. The video includes an introduction of Asport as such, as well as the services. The structure of the video was chosen in such a way that Asport was introduced as such, since it must be assumed that many participants had never heard of Swisscom's product area. Subsequently, the viewers were guided through a video production process from video recording to distribution. Screen recordings of the system were made for the most realistic possible implementation and an example video of an automated recording was shown.

### Quality Criteria

In quantitative studies, the three quality criteria of objectivity, validity, and reliability are relevant and discussed below. In this paper, evaluation objectivity is fulfilled by documenting the applied analysis methods and the data preparation procedure. This allows the results to be understood independently of individuals (Baur and Blasius, 2014). Furthermore, the objectivity of the online survey can be safeguarded, as the respondents answered a standardized survey and were not influenced by an interlocutor (Albers et al., 2009). However, interpretive objectivity cannot be guaranteed in social science research because the assessment of results varies from person to person (Baur and Blasius, 2014).

Validity examines whether or not the survey instrument measures the desired facts (Atteslander et al., 2010), the focus of the three forms of validity testing in this study being construct validity. Since two existing models and their scales were used to test acceptance and willingness to pay, this criterion can be accepted as proven (Baur and Blasius, 2014).

The reliability of measurements shows how dependable they are and is shown when repetition leads to the same results (Atteslander et al., 2010). The consistency and temporal stability of the constructs for testing acceptability were determined by Cronbach's alpha in this study (Baur and Blasius, 2014). In addition, the standardized survey, which is one of the explicit survey methods, provides test-retest reliability (Berekoven et al., 2009). The Van Westendorp method can be assumed to have good implementation and evaluation reliability due to a clearly specified procedure, but this method has a weakness in the reliability of interpretation. Owing to the different price points provided by this method, the results can be interpreted in different ways, and applications for the business world derived from them (Reinecke et al., 2009).

## Results

Data collection—for which 797 Swiss football clubs were contacted by e-mail—was carried out between 30 April and 10 May 2020, and the results were processed online. One hundred and seventy-six complete and usable data sets were generated (response rate = 22.1%), and the IBM SPSS Statistics 26 tool was used for analysis. Of the football club survey participants, the majority were male (92.6%), and the age range extended from birth years 1950 to 1999. The largest number of subjects were born between 1970 and 1979 (25.6%). When asked about their function in the club, respondents could select multiple answers, explaining the high number of responses (238). The questionnaire was most frequently completed by presidents (32.4%), followed by sports directors (16%) and coaches (16%). The table below summarizes the characteristics of the sample. Of the sports clubs surveyed, 25% already produce video recordings; the most common reason for 44.3% of these clubs was to analyze matches, followed by training (22.8%). In addition to the suggested answers, other reasons given were that the recording of matches in this league was compulsory in that regional association or that the video content was used to create highlight clips for online publication.

The majority of clubs said their first team played in the 3rd division league (39.2%), followed by the 4th division league (23.9%). As already mentioned, the two leading Swiss leagues—the Raiffeisen Super League and Brack.ch Challenge League—were not included in the survey. The largest number of respondents stated that their football club had an annual budget of between CHF 100,000 and CHF 250,000 (30.7%). For 18.8% of the clubs, the annual budget was between CHF 50,000 and CHF 100,000. Only two respondents reported having a budget of over CHF 500,000. League affiliation and yearly budget are two factors (among others) examined to determine whether they might influence willingness to pay for a video service (Table 2).

**Table 2:** Demographic sample characteristics.

**Characteristic**	**Characteristics**	**Volume**	**Percentage**
Gender	Male	163	92.60%
	Female	12	6.80%
	Prefer not to say	1	0.60%
	Total	176	100%
Year of birth	1950–1959	15	8.50%
	1960–1969	44	25.00%
	1970–1979	45	25.60%
	1980–1989	43	24.40%
	1990–1999	29	16.50%
	Total	176	100%
Function within the club (multiple answers possible)	President	77	32.40%
	Treasurer	18	7.60%
	Secretary	12	5.00%
	Sports director	38	16.00%
	Coach	38	16.00%
	Employee	29	12.20%
	Player	26	10.90%
	Total	238	100%
League affiliation	9th	7	4.00%
	8th	42	23.90%
	7th	69	39.20%
	6th	30	17.00%
	5th	20	11.40%
	4th	7	4.00%
	3rd	1	0.60%
	Total	176	100%
Budget	Below 20,000 CHF	17	11.20%
	Between 20,000 and 50,000 CHF	19	12.50%
	Between 50,001 and 100,000 CHF	33	21.70%
	Between 100,001 and 250,000 CHF	54	35.50%
	Between 250,001 and 500,000 CHF	27	17.80%
	Above 500,001	2	1.30%
	Total	152	100%
	Missing	24	

Participants were also asked to assess the added value of Asport in five different areas, and a five-point Likert scale from “very little added value” to “very great added value” was used for this purpose. The greatest added value of the system was in developing the performances of the clubs' first teams (*M* = 3.72).

### Reliability Testing of the Constructs

Internal consistency must be established to test whether the individual items of a multi-item scale measure the same thing (Pallant, 2003), and one way to test for this is split-half reliability, which Cronbach's alpha can determine. All constructs except facilitating conditions have a Cronbach's alpha value of over α > 0.7. For facilitating conditions, this is α = 0.53. However, by omitting an item, the value could be increased to α = 0.6. Even when the value lay below the typically required α = 0.7, based on Schmitt (1996), the scale was retained in the model (Table 3).

**Table 3:** Reliability of the constructs.

**Construct**	**Reliability α**
Performance expetancy (PE)	0.77
Effort expetancy (EE)	0.78
Social influence (SI)	0.86
Faciliating conditions (FC)	0.53
Self-efficacy (SE)	0.79
Anxiety (A)	0.77
Attitude to use (ATU)	0.80
Behavioral intention (BI)	0.85

### Influence of Independent Variables

To test the hypotheses from the conceptual model, regression analysis was performed (Field, 2009). Hypotheses H1a, H1b, H1c, H1d, H1e, and H1f state that performance expectancy, effort expectancy, social influence, facilitating conditions, self-efficacy, and anxiety significantly influence attitude to use. A linear regression model was modeled using this dependent variable to test the hypotheses. The independent variables can explain 60% of the variance in attitude to use. The linear regression model is significant [*F*_(6, 169)_ = 42.14, *p* < 0.001]. The three constructs of performance expectancy, effort expectancy, and social influence have a positive significant impact on attitude to use. Here, effort expectancy (β = 0.32, *p* < 0.001) has the strongest influence, performance expectancy (β = 0.31, *p* < 0.001) has the second strongest influence, and social influence (β = 0.28, *p* < 0.001) has the weakest influence. Facilitating conditions, self-efficacy, and anxiety are not significant influencing variables. Hypotheses H1a, H1b, and H1c can therefore be accepted (Table 4).

**Table 4:** Influence of independent variables on attitude to use.

**Model summary^a^**								
**Model**	***R***	***R* ^**2**^**	**Corrected *R*^**2**^**	**SE**	**Durbin-Watson**			
1	0.77^a^	0.60	0.59	0.39	2.06			
**Anova^a^**								
**Model**		**Square-sum**	**Df**	**Mean of the squares**	***F***	**Sig**.		
1	Regression	38.72	6	6.45	42.14	0.000^b^		
	Non-std. residuals	25.89	169	0.15				
	Total	64.61	175					
**Coefficients^a^**								
**Model**		**Non-Std. Coefficients**		**Std. Coefficients**	**T**	**Sig**.	**Collinearity Statistics**	
		**β**	**SE**	**Beta**			**Tolerance**	**VIF**
1	(Constant)	0.54	0.33		1.63	0.104		
	UTAUT_PE	0.20	0.04	0.31	5.17	0	0.67	1.50
	UTAUT_EE	0.34	0.08	0.32	4.29	0	0.42	2.36
	UTAUT_SI	0.24	0.05	0.28	4.76	0	0.67	1.49
	UTAUT_FC	0.09	0.07	0.09	1.41	0.16	0.53	1.88
	UTAUT_SE	0.05	0.05	0.06	1.10	0.27	0.82	1.21
	UTAUT_A	−0.03	0.05	−0.03	−0.53	0.60	0.72	1.40

a *Dependent variable: UTAUT_ATU*.

b *Influencing variables: (constant), UTAUT_PE, UTAUT_EE, UTAUT_SI, UTAUT_FC, UTAUT_SE, UTAUT_A*.

Hypotheses H2a, H2b, H2c, H2d, H2e, and H2f state that performance expectancy, effort expectancy, social influence, self-efficacy, facilitating conditions, and anxiety significantly affect behavioral intention. The independent variables can explain 66% of the variance in behavioral intention. The linear regression model is significant [*F*_(6, 169)_ = 54.97, *p* < 0.001]. The three independent variables performance expectancy, effort expectancy, and social influence have a significant positive impact on behavioral intention. Social influence (β = 0.45, *p* < 0.001) had the strongest influence, performance expectancy (β = 0.33, *p* < 0.001) had the second strongest influence, and effort expectancy (β = 0.20, *p* =0.005) had the weakest influence. The constructs facilitating conditions (β = 0.036, *p* =0.554), self-efficacy (β = 0.09, *p* = 0.082), and anxiety (β = 0.02, *p* = 0.741) were not significant. Consequently, the null hypothesis can be rejected for hypotheses H2a, H2b, and H2c, and the named can be confirmed (Table 5).

**Table 5:** Influence of independent variables on behavioral intention.

**Model summary^a^**								
**Model**	***R***	***R* ^**2**^**	**Corrected R2**	**SE**	**Durbin-Watson**			
1	0.81	0.66	0.65	0.51	2.14			
**Anova^a^**								
**Model**		**Square-sum**	**Df**	**Mean of the squares**	***F***	**Sig**.		
1	Regression	84.63	6	14.11	54.97	0		
	Non-std. residuals	43.36	169	0.26				
	Total	128.00	175					
**Coefficients^a^**								
**Model**		**Non-Std. coefficients**		**Std. coefficients**	**T**	**Sig**.	**Collinearity statistics**	
		***β***	**SE**	**Beta**			**Tolerance**	**VIF**
1	(constant)	−1.15	0.43		−2.68	0.01		
	UTAUT_PE	0.30	0.05	0.33	6.07	0	0.67	1.50
	UTAUT_EE	0.29	0.10	0.20	2.83	0.01	0.42	2.36
	UTAUT_SI	0.53	0.06	0.45	8.32	0	0.67	1.49
	UTAUT_FC	0.05	0.08	0.04	0.59	0.55	0.53	1.88
	UTAUT_SE	0.11	0.06	0.09	1.75	0.08	0.82	1.21
	UTAUT_A	0.02	0.07	0.02	0.33	0.74	0.72	1.40

a *Dependent variable: UTAUT_BI*.

Hypothesis H3 states that attitude to use has a significant influence on behavioral intention. This was tested below by the linear regression model. The independent variable can explain 58% of the variance of the dependent variable. The model is significant [*F*_(1, 174)_ = 235, *p* < 0.001]. Attitude to use has a positive significant effect on behavioral intention (β = 1.07, *p* =0.001). Moreover, the BCa confidence interval obtained by the bootstrapping procedure does not include the value zero, and accordingly, the result is robust (Urban and Mayerl, 2018). Hypothesis H3 can therefore be confirmed (Table 6).

**Table 6:** Influence of attitude to use on behavioral intention.

**Model summary^a^**							
**Model**	***R***	***R* ^**2**^**	**Corrected *R*^**2**^**	**SE**	**Durbin-Watson**		
1	0.76	0.58	0.57	0.56	1.8		
**Anova^a^**							
**Model**		**Square-sum**	**df**	**Mean of the squares**	***F***	**Sig**.	
1	Regression	73.54	1	73.54	235.00	0	
	Non-std. residuals	54.45	174	0.31			
	Total	128.00	175				
**Bootstrap for coefficients^a^**							
**Model**		***β***	**Bootstrap**				
		**Distortion**	**SE**	**Sig. (2-sided)**	**BCa 95% confidence interval**		
					**Lower value**	**Upper Value**	
1	(Constant)	−0.64	0.01	0.25	0.01	−1.17	−0.12
	UTAUT_ATU	1.07	−0.00	0.07	0.00	0.94	1.18

a *Dependent variable: UTAUT_BI*.

Hypothesis H4 tests whether attitude to use has a significant impact on willingness to pay. The independent variable can explain 2.5% of the variance of the dependent variable. The regression model is significant [*F*_(1, 174)_ = 4.48, *p* = 0.036]. Attitude to use has a positive significant effect on willingness to pay (β= 387.461, *p* = 0.022). The model is robust because the BCa confidence interval does not include the value zero. Hypothesis H4 is therefore confirmed (Table 7).

**Table 7:** Influence of attitude to use on willingness to pay.

**Model summary^a^**							
**Model**	***R***	***R* ^**2**^**	**Corrected *R*^**2**^**	**SE**	**Durbin-Watson**		
1	0.16	0.03	0.02	1,472.16	1.74		
**Anova^a^**							
**Model**		**Square-sum**	**Df**	**Mean of the squares**	***F***	**Sig**.	
1	Regression	9,699,275.3	1	9,699,275.3	4.48	0.04	
	Non-std. residuals	377,101,043	174	2,167,247.4			
	Total	386,800,318	175				
**Bootstrap for coefficients^a^**							
**Model**	***β***		**Bootstrap**				
			**Distortion**	**SE**	**Sig**.	**BCa 95% confidence interval**	
					**(2-sided)**	**Lower value**	**Upper Value**
1	(Constant)	57.52	9.85	658.43	0.93	−1,123.02	1,329.58
	UTAUT_ATU	387.46	−2.02	178.27	0.02	50.84	719.45

a *Dependent variable: UTAUT_WTP*.

Hypothesis H5 tests whether behavioral intention has a significant impact on willingness to pay. The independent variable can explain 1% of the variance of the dependent variable. The regression model is not significant [*F*_(1, 174)_ = 1.79, *p* < 0.183]. Therefore, the influence of behavioral intention on willingness to pay is also not significant (β = 175.29, *p* = 0.147). This is also confirmed by the BCa confidence interval, which passes through the zero point. Hypothesis H5 is therefore rejected (Table 8).

**Table 8:** Influence of behavioral intention on willingness to pay.

**Model summary^a^**							
**Model**	***R***	***R* ^**2**^**	**Corrected *R*^**2**^**	**SE**	**Durbin-Watson**		
1	0.10	0.01	0.00	1,483.37	1.74		
**Anova^a^**							
**Model**		**Square-sum**	**df**	**Mean of the squares**	***F***	**Sig**.	
1	Regression	3,932,864.3	1	3,932,864.3	1.79	0.18	
	Non-std. residuals	382,867,454.2	174	2,200,387.7			
	Total	386,800,318.5	175				
**Bootstrap for coefficients^a^**							
**Model**		***β***	**Bootstrap**				
			**Distortion**	**SE**	**Sig**.	**BCa 95% confidence interval**	
					**(2-sided)**	**Lower value**	**Upper value**
1	(Constant)	917.26	−4.21	398.75	0.02	132.39	1,672.39
	UTAUT_BI	175.30	1.59	116.46	0.15	−35.02	409.34

a *Dependent variable: UTAUT_WTP*.

### Influence of the Moderators on Attitude to Use

Hypotheses H6a,b,c,d,e,f and H7a,b,c,d,e,f test whether the relationship between the independent variables and attitude to use is affected by age or gender. For the moderator analysis, we used the SPSS macro PROCESS (Version 3.5) by Andrew Hayes, which is considered to be particularly strong for testing (Baltes-Götz, 2018). The following table summarizes the results of the analyses for the moderator variables of age and gender (Table 9).

**Table 9:** Influence of moderators on attitude to use.

**Designation**	***R* ^**2**^**	**df**	***F***	**Sig. (model)**	**Sig. (interaction-effect)**	**Result**
H6a: PE × Age	0.34	3/172	27.88	<0.001	0.09	–
H6b: EE × Age	0.37	3/172	24.91	<0.001	0.11	–
H6c: SI × Age	0.35	3/172	35.52	<0.001	0.12	–
H6d: FC × Age	0.27	3/172	19.72	<0.001	0.03	+
H6e: SE × Age	0.13	3/172	6.63	<0.001	0.05	–
H6f: A × Age	0.11	3/172	5.56	<0.05	0.07	–
H7a: PE × Gender	0.33	3/172	26.46	<0.001	0.53	–
H7b: EE × Gender	0.35	3/172	23.99	<0.001	0.89	–
H7c: SI × Gender	0.33	3/172	31.12	<0.001	0.58	–
H7d: FC × Gender	0.24	3/172	13.41	<0.001	0.40	–
H7e: SE × Gender	0.11	3/172	6.22	<0.001	0.89	–
H7f: A × Gender	0.11	3/172	4.87	<0.05	0.33	–

The table shows that the models are significant in each case, but the interaction effect between the individual independent variables and age and gender is not. Only in the relationship between facilitating conditions and attitude to use was a significant age influence found. The model is significant and explains 27% of the variance [*F*_(3, 172)_ = 19.72, *p* < 0.0001]. The interaction effect is significant (*p* = 0.0258), and the results show that as the age variable increases, the effect also becomes stronger. Age as a variable was questioned by asking the year-of-birth range. Accordingly, the effect of age on attitude to uses increases the younger the users are. Hypothesis H6d is therefore accepted, and the remaining hypotheses H6 and H7 are rejected.

### Influence of Moderators on Behavioral Intention

Hypotheses H8a,b,c,d,e,f and H9a,b,c,d,e,f test whether the relationship between the independent variables and behavioral intention is affected by age or gender. The PROCESS macro was again used for the calculation. The following table summarizes the results of the analyses for the moderator variables of age and gender (Table 10).

**Table 10:** Influence of moderators on behavioral intention.

**Designation**	**R2**	**Df**	***F***	**Sig. (model)**	**Sig. (interaction-effect)**	**Result**
H8a: PE × Age	0.43	3/172	45.73	<0.001	0.04	+
H8b: EE × Age	0.23	3/172	14.33	<0.001	0.23	–
H8c: SI × Age	0.50	3/172	91.07	<0.001	0.41	–
H8d: FC × Age	0.16	3/172	8.89	<0.001	0.14	–
H8e: SE × Age	0.11	3/172	5.49	<0.05	0.20	–
H8f: A × Age	0.04	3/172	2.19	0.09	0.87	–
H9a: PE × Gender	0.42	3/172	40.85	<0.001	0.49	–
H9b: EE × Gender	0.22	3/172	14.71	<0.001	0.58	–
H9c: SI × Gender	0.50	3/172	84.69	<0.001	0.15	–
H9d: FC × Gender	0.14	3/172	8.28	<0.001	0.34	–
H9e: SE × Gender	0.09	3/172	4.48	<0.05	0.77	–
H9f: A × Gender	0.04	3/172	2.26	0.08	0.95	–

The table shows that most of the models are significant in each case, but the interaction effect between the individual independent variables and age and gender is not. For both the moderator age and gender, the model is not significant for the independent variable fear [age: *F*_(3, 172)_ = 2.19, *p* = 0.0909; gender: *F*_(3, 172)_ = 2.26, *p* = 0.0831]. Only for the independent variable performance expectancy and the moderator age is both the model [*F*_(3, 172)_ = 45.73, *p* < 0.0001] and the interaction effect significant (*p* = 0.0409). The evaluation shows that the effect, in turn, also becomes stronger with an increase in age. Age as a variable was questioned by asking the year-of-birth range. Accordingly, the effect of age on performance expectancy increases the younger the users are. Hypothesis H8a is therefore accepted, and the remaining hypotheses of H8 and H9 are rejected.

### Willingness to Pay

Willingness to pay was tested using the Van Westendorp method (1976). In addition to the use of values in the conceptual model, the classical analysis of this model is presented below. In the survey, subjects were asked to answer the four willingness-to-pay questions. The annual license price for Asport, including the automated camera system, the content management system, and the video portal, had to be quantified. In our evaluation, a price range of CHF 500 was initially defined, and then the frequency distributions per question were calculated using Microsoft Excel. To map the results, these frequencies were cumulated on a percentage basis. The questions resulted in the four curves: “too expensive,” “expensive,” “cheap,” and “too cheap.” These depict willingness to pay in relation to percentage frequency. As described in the theory section, in preparation for the evaluation, the inverses of the two graphs “expensive” and “cheap” were formed and labeled “not expensive” and “not cheap.”

**Figure 2:**
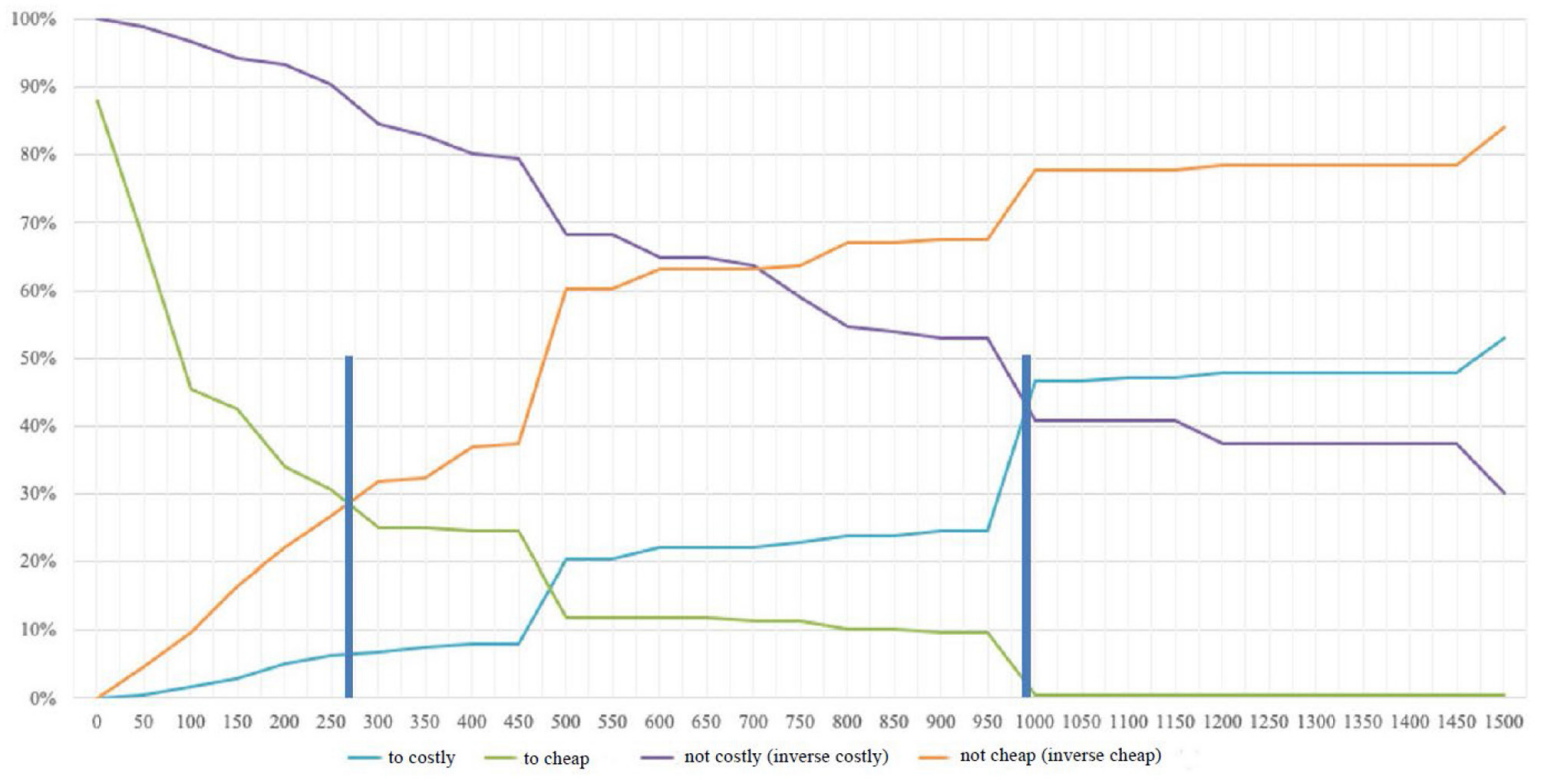
Determination of the acceptable price range according to Van Westendorp (1976).

Part of Van Westendorp's analysis is to define the acceptable price range for a product or service. To determine the acceptable price range, the inverses of the “favorable” and “expensive” curves are formed and defined as “not favorable” and “not expensive” (Reinecke et al., 2009). The intersection of the two curves “not cheap” and “too cheap” is chosen as the lower price limit. However, this does make sense, as an additional reduction in price would lead to the proportion of those who consider the product to be too favorable exceeding the proportion of those who consider it not favorable (Reinecke et al., 2009). The price ceiling is determined by the intersection of the “too expensive” and “not expensive” curves and is also described as the point of marginal expensiveness. An increase in price makes no sense since in that case, the proportion of people who would describe the product as too expensive exceeds those who see the product as not expensive (Reinecke et al., 2009). The lower price limit, indicated by the bar, is determined by the intersection of the “not cheap” and “too cheap” curves at CHF 270. The upper price limit is shown by the intersection of the “not expensive” and “too expensive” curves at CHF 990 (Figure 2).

**Figure 3:**
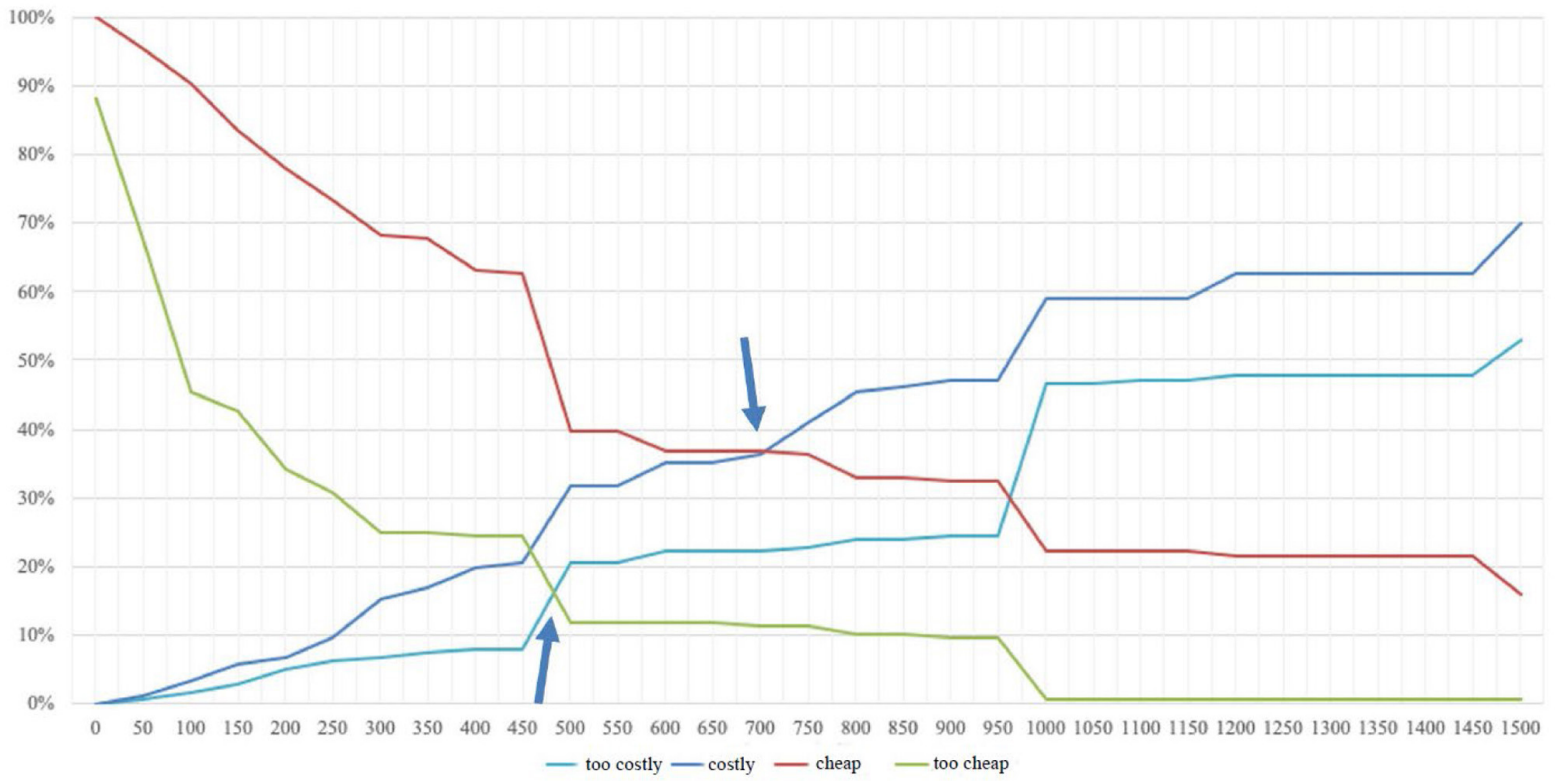
Determination of the optimal price point according to Van Westendorp (1976).

In addition to the acceptable price range, the method also offers guidance on the optimal price point. The optimum price point is defined by the intersection of the two curves “too cheap” and “too expensive.” In this case, the same number of potential customers indicates that they perceive the product as too cheap or too expensive (Khandker and Joshi, 2019). This score does not take into account cost structures but shows the optimal price from the perspective of the customer. This is the case when customer resistance to purchase is at its lowest (Lewis and Shoemaker, 1997). The indifference price is formed from the curves “expensive” and “cheap” and represents the fact that at this price an equal number of respondents state that it is expensive or cheap (Khandker and Joshi, 2019). According to Van Westendorp, this price usually represents the median price that customers effectively pay or that of the market leader (1976). Moreover, the difference between the indifference price and the optimal price shows the price sensitivity of potential customers. The smaller the difference, the more sensitive (Reinecke et al., 2009). The figure below shows that the optimal price point lies at the intersection of the “too expensive” and “too cheap” curves at CHF 485. The indifference point can also be determined based on the intersection of the “cheap” and “expensive” graphs at CHF 700 (Figure 3).

When all three independent variables are included, it is shown that Levene's test is significant [*F*_(23, 124)_ = 1.83, *p* = 0.019] and, accordingly, variance homogeneity cannot be assumed. An additional test shows that when the independent variable budget is excluded, the Levene test is no longer significant [*F*_(7, 168)_ = 0.87, *p* = 0.532] and the conditions are met. Accordingly, the results of the two-factor ANOVA with the independent variables league and video are presented below first, followed by a one-factor ANOVA with the independent variable budget. The two-factor analysis of variance tests whether there is a significant difference in the assessment of willingness to pay depending on which league a club plays in and whether a club already produces video recordings. It is found that the overall model is not significant [*F*_(7, 168)_ = 0.45, *p* = 0.866] and accordingly there is no significant difference in the assessment. As noted, by using the Welch test, the test using the single factor ANOVA of whether there is a significant difference in the assessment of willingness to pay, depending on how much the club's budget is, is possible. This is despite the fact that Levene's test shows significant values [*F*_(3, 148)_ = 3.87, *p* = 0.011]. The results of Welch's test show that there is a significant difference in the assessment [*F*_(3, 73)_ = 2.78, *p* = 0.047]. To find out which factors are different, a *post-hoc* test is conducted using Games-Howell procedure (IBM, 2014). There is a significant difference (*p* =0.049) in the assessment of willingness to pay between clubs that have a budget below CHF 50,000 (*M* = 1040.53, SD = 1120.40) and those that have a budget above CHF 250,000 (*M* = 2215.45, SD = 2127.29).

## Discussion

The following sub-chapters are structured according to the conceptual model and the hypotheses derived from it, with the moderator analysis integrated into the respective chapters. To conclude, the willingness to pay findings are discussed independently of the conceptual model.

Hypotheses H1a,b,c,d,e,f postulate a direct significant influence of performance expectancy, effort expectancy, social influence, facilitating conditions, self-efficacy, and anxiety on attitude to use. The results of the study show that the hypotheses H1a, H1b, and H1c can be confirmed. In the basic model of Venkatesh et al. (2003), the direct influences of the independent variables on attitude to use were not tested, but only that of attitude to use on behavioral intention. In contrast, the meta-analysis by Dwivedi et al. (2019) also examined the direct influence on attitudes toward use. However, the two independent variables self-efficacy and anxiety were omitted. The meta-analysis concluded that the strongest influencing factor was performance expectancy, followed by effort expectancy and social influence (Dwivedi et al., 2019). This contrasts with this study, in which social influence is also the weakest factor, but effort expectancy has a stronger effect than performance expectancy.

Moderator analysis to test hypotheses H6 and H7 with the sub-hypotheses shows only one significant influence of a moderator on the relationship between the independent variables and attitude to use. Hypothesis H6d is accepted because age influences the effect between facilitating conditions and attitude to use. Moreover, the influence of the independent variables becomes significant only through the influence of the moderator. This effect is further amplified in younger people. The relationship between facilitating conditions and attitude to use is also evidenced by Dwivedi et al. (2019) but without a moderator effect, as these were not evaluated. The fact that most moderator-related constructs were rejected (H6-H9) is probably due to the fact that age and gender are less important in the use of ICT in a professional context than, for example, in the context of social media in individuals' leisure time. Employees and managers are accustomed to using certain ICT, whether the employees are younger or older or male or female. However, the gender-specific result should be treated with caution due to the high number of male participants (92.6%).

Hypotheses H2a,b,c,d,e,f postulate a significant influence of performance expectancy, effort expectancy, social influence, facilitating conditions, self-efficacy, and anxiety on behavioral intention. As reported in the results section of this paper, hypotheses H2a, H2b, and H2c were accepted. Unlike the dependent variable attitude to use, social influence has the strongest effect, followed by performance expectancy and effort expectancy. The impact strength of the determinants also differs compared with the original model. The results from Venkatesh et al. (2003) show that social influence was significant only by moderators, whereas in this study, the two moderators tested (age and gender) show no interaction effect. Performance expectancy was the strongest factor in the initiators' UTAUT model, and the effect was greater for males and younger individuals (Venkatesh et al., 2003). A moderator effect of age but not for gender was also found in this study. In contrast to the study by Venkatesh et al. (2003), a significant relationship was also found for effort expectancy, but with no moderator effect. The results of facilitating conditions, self-efficacy, and anxiety are consistent with those of the original study, and no significance was demonstrated in either study (Venkatesh et al., 2003). These findings differ from the meta-analysis by Khechine et al. (2016), which demonstrated an influence on behavioral intention in facilitating conditions. In this paper, behavioral intention is equated with acceptance of the system, and the results show that 66% of the variance can be explained. The result is similar to Venkatesh et al. (2003), who could explain 70% of behavioral intention. The null hypothesis was rejected for hypotheses H2a, H2b and H2c, as the constructs facilitating conditions, self-efficacy and anxiety were not significant, in contrast to Venkatesh et al. (2003). One reason might be that our study surveyed club managers rather than end consumers. The use of ICT is common in a business context, which is one reason why the constructs did not show significance.

Hypothesis H3 tested whether there was a significant effect of attitude to use on behavioral intention. This effect could be demonstrated, and the hypothesis was confirmed. However, the literature shows contradictory results. No significance was demonstrated by Venkatesh et al. (2003) but it was in the meta-analysis by Dwivedi et al. (2019).

Hypotheses H4 and H5 tested whether there was a significant influence of the variables attitude to use and behavioral intention on willingness to pay. It was shown that such an effect could only be demonstrated for attitude to use. However, the level that could explain this relationship was low at 2.5%, showing that other determinants explain much of the variance in willingness to pay—but these were not part of this study. While studies could be found in the literature that have inserted willingness to pay in some form into technology acceptance models such as TAM or UTAUT, there is no standard implementation variant. However, it can be said that significant effects have also been demonstrated in the literature, for example, in Lee et al. (2015), which adapted the TAM and showed that customer attitudes affect willingness to pay. Another study again chose willingness to pay as the study endpoint but omitted attitude to use and behavioral intention (Stephanidis, 2019). The results showed that performance expectancy and facilitating conditions as variables from UTAUT, as well as trust and long-tail effects, have an impact on willingness to pay. This explained the 67% variance and, accordingly, significantly more than in the present paper with a variable attitude to use (Stephanidis, 2019). That the influence of behavioral intention on willingness to pay is not significant and the null hypothesis can therefore be rejected for hypotheses H2a is probably caused by the fact that the benefits for the user to use Asport were not clearly evident.

The results of willingness to pay according to the Van Westendorp method show that the price range for the three service components of Asport starts at CHF 270 per year, rising to CHF 990. According to Reinecke et al. (2009), it is precisely this price range that is relevant for innovative services where price expectations do not yet exist, as is the case of Asport. With this knowledge and the optimal price point (which is lower than the upper point of the price range), Asport's pricing can be based on willingness to pay. If we compare the results with the current prices offered by Asport, we find that they are much higher than the sports clubs' willingness to pay. If one disregards the initialization fee, which the club must pay just once, the club will spend at least CHF 2,900 per year (Asport, 2020). The difference from the upper price limit is CHF 1,910, which is a high discrepancy at this relatively low-price level. Even more significant is the difference from the indifference point, at CHF 700. The optimal price point, which indicates the lowest resistance to purchase, was determined at CHF 485—a difference of CHF 2,415 from the Asport price. In other words, willingness to pay is 83.3% lower than the asking price. However, variations exist in the assessment of willingness to pay. For example, it has been demonstrated that clubs with a budget of less than CHF 50,000 are significantly less willing to pay than those with a budget of over CHF 250,000.

### Implications for Theory

When considering what contribution this paper makes to state-of-the-art technology acceptance research, several aspects need to be noted. There are thousands of studies that have applied or adapted the UTAUT model. Consequently and according to Shachak et al. (2019), a high level of knowledge has been achieved in this research area. Due to some consistency in the results, the new explanatory content is explicit. However, some studies apply technology acceptance in the context of sports technologies (among others: Hur et al., 2011; Chien-Ta and Chao-Hsiang, 2015), but none examines a comparable service with this level of functionality. In addition, the studies identified often focus on the private end-consumer rather than a sports club (among others: Kwak and McDaniel, 2011; Ibrahim, 2014; Byun et al., 2018; Kim and Chiu, 2019). The findings discussed in the previous chapter also show that deviations from the basic model could be identified, for example, that there is a direct relationship between the independent variables attitude to use and further to behavioral intention or regarding the strongest drivers acting on behavioral intention. Another aspect is the integration of the willingness to pay variable into the UTAUT model. As mentioned above, although there are studies in which willingness to pay plays a role in technology acceptance models, none can be considered a standard implementation. Accordingly, this paper contributes to this specific area and through the application of the evaluation method. This is because it was possible to prove that attitude to use has an influence, even though the explanatory power is low at 2.5%. In summary, based on the reasons listed, a contribution to research could be made in this context.

### Implications for Asport and Sports Industry

This study shows the relevant factors that lead to behavioral intention or acceptance of Asport services among amateur football clubs in German-speaking Switzerland. The three independent variables of social influence, performance expectancy, and effort expectancy are all important, as is attitude to use. Although acceptance does not influence willingness to pay, it does influence attitudes to use, which underlines the relevance of this factor. Accordingly, the facilitating conditions, which have an additional effect on attitude to use through the moderator age, must also be considered. The information gained helps to set the right focus, such as product design or communication, to influence and improve user acceptance through targeted measures.

Social influence emerges as the most decisive factor in acceptance. This means that it is people in both the private and club environment who greatly influence whether the system is used or not. One way to use this influencing factor to your advantage is to either target opinion leaders or make the result of the product itself somehow accessible and shareable to the mass. In the case of targeting opinion leaders, the aim must be to get people with a lot of influence to be positive about buying and using the system, and to talk about it. From Asport's perspective, this can be beneficial on two levels. Once the sports club has obtained the system (and the aim is to get as many potential users as possible to buy it), an attempt can be made to accelerate this process via opinion leaders and win over other sports clubs as customers. If opinion leaders in the form of associations or other sports clubs in the region use the system and talk about it, this can boost sales. In the case of making the product itself somehow accessible and shareable to the fans of the clubs, the aim must be to integrate some kind of shareability function from Asport to social media platforms like Instagram, YouTube and TikTok. From Asport's perspective, this can be beneficial on two levels. The distribution of content snippets produced thanks to Asport's technology enhance the company's goal of making clubs more attractive to potential fans, players and club contributors. If other club officials see these content snippets, it raises the brand awareness of Asport as a brand and therefore will improve its standing in sales negotiations. For both cases, integration of opinion leaders to the marketing mix and shareability of the content produced, club environments and especially their management must comprehend the market stakeholders and know the personas contained by it, in order to be able to decide upon the right opinion leaders characteristics and content distribution platforms to work with. This development opens up the potential for smaller marketing agencies focusing on amateur sports clubs.

To meet the user performance expectancy and promote acceptance, services must offer added value in the club's day-to-day activities. It is important that tasks can be completed more quickly, and the system seen as useful. To achieve this, the precise needs of the user must be understood. Our findings showed that the sports clubs surveyed saw the greatest added value in the development of sporting performance. Furthermore, clubs that already produce video recordings use them mainly to analyze matches in training sessions. This shows that the focus of the clubs is on their sport. Asport can use this information to optimize and further develop the product, emphasizing these benefits. A priority should be placed on younger people, as they place particular value on system performance. However, other areas of added value, such as marketing, should not be overlooked. Since Asport offers hitherto unknown new possibilities for clubs, there is still a lack of empirical evidence with which to evaluate these potential benefits. This should be considered when communicating to the public, for example, through the use of reference projects, enabling added value to be accessible and understandable when implementing the system at a sports club. For the sports industry this highlights the need of clarification of anticipation of application's expectations. This will also have an influence on future human resource planning and recruitment. Future management officials in the sports industry will have to understand technological advancements, in order to be able to take into consideration or even use new services such as Asport. This will have implications in sports management education as well.

Another aspect that could help Asport gain acceptance from users concerns effort expectancy. Services must be as straightforward, clear, and comprehensible as possible. It is recommended that Asport works with users to identify potential problems or barriers to use and feed these back into product design. It is also important that learning how to use the system is as simple as possible and requires minimal effort. One option would be to offer help in the form of online tutorials, which explain the use of the system through videos or webinars. Here, the key functions are presented, and users can interactively discuss any challenges they have faced.

The area of facilitating conditions is complex, and an essential aspect is that the prerequisites for using Asport are as low as possible. Since most people now have several digital devices, online access is assumed. Nevertheless, the system should be compatible with existing systems in everyday use, for example, if clubs are already using analytic tools. Since Asport is an open system, interfaces can offer the necessary links (Asport, 2020). One possible acceptance lever would be to guarantee that users can get help quickly and easily when issues arise, whether through direct contact with Asport or via an online community platform. Since the influence of facilitating conditions on attitude to use only becomes relevant through the moderator of age and the effect is intensified in younger people, the requirements of this target group should be given particular attention when analyzing and implementing features.

Evaluation of willingness to pay by football clubs has shown that this is significantly lower than the price offered by Asport. However, it would be presumptuous to deduce that the price should be reduced to that level; instead, willingness to pay should be investigated in greater detail. Notwithstanding, the financial possibilities of a sports club in the amateur sector are limited, and accordingly, the price ultimately determines whether technology is purchased or not (Volkmann et al., 2019). This is also confirmed in the findings since sports clubs with higher budgets show a greater willingness to pay. Of course, it could be argued that the first step is to identify sports clubs with higher budgets and focus sales efforts on them. However, there might be better and more inclusive ways to make Asport, still, a service for amateur clubs, too. One logical way would be to demonstrate how sports clubs can benefit financially from Asport. Points in favor of purchasing Asport can be created if the clubs receive empirical figures based on reference projects about how much additional money they could earn through digital marketing, especially when this amount exceeds the annual price in the best-case scenario. At the same time, it should be determined which functions the clubs perceive as benefits and how this affects their willingness to pay. Another way to stay more inclusive with pricing, would be to find and test additional pricing tactics for the services offered by Asport, which in turn could be revolutionary for the sports industry, for example trying to introduce dynamic pricing based on the incremental revenues obtained thanks to the use of the technology itself. These methods can be tested using conjoint analysis and provide essential clues for development and communication focus (Völckner, 2006). These measures must positively influence willingness to pay through targeted product design and, above all, communication with potential customers. Owing to the novelty of these services, such public relations activity is paramount.

### Limitations and Outlook

This study has limitations that must be considered when interpreting the results and recommendations. One limitation is that the participants in the survey were probably unaware of the Asport system and certainly had never used it before. Since effective prior use was not possible in this research, a 3-min video was created to introduce Asport and its features. First, this may have made it difficult to answer the questions based on the constructs of the UTAUT model. Second, the subjects may not have fully understood the added values and benefits of the system—or the video may not have explained them sufficiently. Therefore, it is possible that our assessment of willingness to pay was adversely affected, producing the resulting low values and effective prices.

Another potentially critical area is the evaluation procedure. When evaluating social and behavioral science research questions, multiple regression analysis and structural equation modeling are appropriate (Kupper, 1997). The structural equation model is considered more robust, and it can be analyzed more comprehensively since direct relationships as well as an entire model can be assessed (Kupper, 1997). This limitation has been shown, among other things, in the handling of moderators. Due to the limited possibilities of the PROCESS macro in SPSS, these could only be considered in isolation. This means that only the interaction effect of one independent variable and one moderator on the dependent variable could be tested at a time.

In a future study and for a deeper understanding of acceptance, the survey would need to be conducted among actual Asport users rather than potential users. This would prevent people who have no contact with the system whatsoever from answering the questionnaire, as was possible in this study. In this case, such participants were asked to answer from the perspective of the club as a whole. Furthermore, the survey should not have been conducted in one session but several times in succession. In this way, the experience and learning effect could have be verified (Venkatesh et al., 2012). In such a study, in which actual users are interviewed, not only the factors that lead to acceptance should be analyzed, but also whether these people show acceptance, i.e., accept the use of the system. This is because a meaningful statement about whether acceptance exists can only be made through repeated use over time (Kollmann, 1998), and this brings the desired added value in practice. For the generalizability of the results and to make statements about amateur sport as a whole, clubs other than football clubs must be included in future studies.

When asking about willingness to pay, a limitation was created by our sample size. Reinecke et al. (2009) state that for a reliable statement, the rule of thumb is for at least 300 subjects to participate in the survey. This study did not achieve that number. As mentioned earlier, further information regarding willingness to pay is of interest. However, from a theoretical point of view, it is necessary to determine which factors influence the willingness to pay and—from the company's point of view—it is relevant to find out what benefit components influence the assessment to what extent.

In this paper, the focus has been on video-related services for football clubs in Switzerland. It will be fascinating to see how this market develops and to what extent, for example, automated camera systems will become standard in the future. However, the opportunities for Asport and other companies in the sports technology market can influence many other stakeholders. Whether and to what extent the currently existing structures and roles of media, sponsors, or clubs will change because of the possibilities offered by this technology remains to be seen.

## Data Availability Statement

The raw data supporting the conclusions of this article will be made available by the authors, without undue reservation.

## Ethics Statement

Ethical review and approval was not required for the study on human participants in accordance with the local legislation and institutional requirements. The patients/participants provided their written informed consent to participate in this study.

## Author Contributions

MH, FH, and VS contributed to conception and design of the study and wrote sections of the manuscript. MH organized the database. FH performed the statistical analysis. MH wrote the first draft of the manuscript. All authors contributed to manuscript revision, read, and approved the submitted version.

## Conflict of Interest

FH was employed by the company Swisscom Broadcast AG. The remaining authors declare that the research was conducted in the absence of any commercial or financial relationships that could be construed as a potential conflict of interest.

## Publisher's Note

All claims expressed in this article are solely those of the authors and do not necessarily represent those of their affiliated organizations, or those of the publisher, the editors and the reviewers. Any product that may be evaluated in this article, or claim that may be made by its manufacturer, is not guaranteed or endorsed by the publisher.
